# Supplementary material for: Impact of productive safety net program on food security of beneficiary households in western Ethiopia: A matching estimator approach
Source: PLoS One. 2022 Jan 21;17(1):e0260817. doi: 10.1371/journal.pone.0260817 (PMC8782465; doi:10.1371/journal.pone.0260817)
Supplement: Attachment — Submitted filename: Response to reviewer.docx [file pone.0260817.s003.docx]

PLOS one response to reviewers

1. Please ensure that your manuscript meets PLOS ONE's style requirements, including those for file naming.

**Response:** The authors tried to meet the PLOS ONE’s style requirements.

2. We suggest you thoroughly copyedit your manuscript for language usage, spelling, and grammar. If you do not know anyone who can help you do this, you may wish to consider employing a professional scientific editing service.

**Response:** The authors has been submitted to professional editors and got revised manuscript

. Address of the editor:

ABOLARIN Gbminiyi, Ph.D.

[abolaring@babcock.edu.ng](mailto:abolaring@babcock.edu.ng)

Editor, College of Postgraduate Studies,

Babcock University, Ogun State, Nigeria

3. PLOS requires an ORCID iD for the corresponding author in Editorial Manager

**Response:**

ORCID-Aregash Getachew Hailu

[**https://orcid.org/0000-0002-4822-0470**](https://orcid.org/0000-0002-4822-0470)

ORCID-Zerihun Yohannes Amare

https://orcid.org/0000-0002-4503-6790

●             The name of the colleague or the details of the professional service that edited your manuscript: **see the full details of the professional editor**

●             A copy of your manuscript showing your changes by either highlighting them or using track changes (uploaded as a *supporting information* file)---**Attached**

●             A clean copy of the edited manuscript (uploaded as the new *manuscript* file)

**Response:  Attached**

1. We note you have included a table to which you do not refer in the text of your manuscript. Please ensure that you refer to Table 3, 10,  12, in your text; if accepted, production will need this reference to link the reader to the Table.

Response: **Corrected**

2. Please ensure that you refer to Figure 1 in your text as, if accepted, production will need this reference to link the reader to the figure.

Response: **Corrected**

6. Please review your reference list to ensure that it is complete and correct. If you have cited papers that have been retracted, please include the rationale for doing so in the manuscript text, or remove these references and replace them with relevant current references. **Any changes to** the reference list should be mentioned in the rebuttal letter that accompanies your revised manuscript. If you need to cite a retracted article, indicate the article’s retracted status in the References list and also include a citation and full reference for the retraction notice.

Response: corrected

7. Thank you for submitting the above manuscript to PLOS ONE. During our internal evaluation of the manuscript, we found significant text overlap between your submission and the following previously published works:

We would like to make you aware that copying extracts from previous publications, especially outside the methods section, word-for-word is unacceptable. In addition, the reproduction of text from published reports has implications for the copyright that may apply to the publications.

Please revise the manuscript to rephrase the duplicated text, cite your sources, and provide details as to how the current manuscript advances on previous work. Please note that further consideration is dependent on the submission of a manuscript that addresses these concerns about the overlap in text with published work.

Response:

We will carefully review your manuscript upon resubmission, so please ensure that your revision is thorough.

Reviewers' comments:

Reviewer's Responses to Questions

**Comments to the Author**

1. Is the manuscript technically sound, and do the data support the conclusions?

The manuscript must describe a technically sound piece of scientific research with data that supports the conclusions. Experiments must have been conducted rigorously, with appropriate controls, replication, and sample sizes. The conclusions must be drawn appropriately based on the data presented.

Reviewer #1: Yes

Reviewer #2: Yes

Reviewer #3: Yes

2. Has the statistical analysis been performed appropriately and rigorously?

Reviewer #1: Yes

Reviewer #2: Yes

Reviewer #3: Yes

3. Have the authors made all data underlying the findings in their manuscript fully available?

The [PLOS Data policy](http://www.plosone.org/static/policies.action#sharing) requires authors to make all data underlying the findings described in their manuscript fully available without restriction, with rare exception (please refer to the Data Availability Statement in the manuscript PDF file). The data should be provided as part of the manuscript or its supporting information, or deposited to a public repository. For example, in addition to summary statistics, the data points behind means, medians and variance measures should be available. If there are restrictions on publicly sharing data—e.g. participant privacy or use of data from a third party—those must be specified.

Reviewer #1: Yes

Reviewer #2: Yes

Reviewer #3: Yes

4. Is the manuscript presented in an intelligible fashion and written in standard English?

PLOS ONE does not copyedit accepted manuscripts, so the language in submitted articles must be clear, correct, and unambiguous. Any typographical or grammatical errors should be corrected at revision, so please note any specific errors here.

Reviewer #1: Yes

Reviewer #2: No

Reviewer #3: No

**Response:**  The language is edited as to the level of standard with accredited editor. The address is described at the beginning of this document.

5. Review Comments to the Author

Please use the space provided to explain your answers to the questions above. You may also include additional comments for the author, including concerns about dual publication, research ethics, or publication ethics. (Please upload your review as an attachment if it exceeds 20,000 characters)

Reviewer #1: The topic of is interesting and relevant. The elaboration and the applied methodology is accurate, however a formal deficiency has to be highlighted: at the tables and figures there are no source indication which is a necessary requirement in case of scientific papers. It can be seen that most probably all (or the majority) of data derived from the primary research of the authors, but even if so, it has to be indicated accordingly.

**Response:** proofread and Corrected:
The bibliographic review could be a bit broadened especially a part of the timely issues and challenges of food supply security in Ethiopia and possibly in some other sub-Saharan African countries to make the context more international. The international bibliography is rich in this respect (e.g. <https://pubs.iied.org/sites/default/files/pdfs/migrate/14640IIED.pdf> or <https://re.volsu.ru/eng/contacts/8_Nad_i_dr.pmd.pdf> .
The authors are also advised to specify and underline the applicability of their new results and findings possibly in a structured list of recommendations. After the suggested improvements were made, the paper can be recommended to be accepted and published.

**Response:** the suggested and other literatures are included

Reviewer #2: The manuscript is technically sound, the data the authors have collected supports the study and the conclusions, thus the study presents the results of original research. What was especially welcomed is the critical approach towards similar program evaluations in Ethiopia and the fact that these previous studies did not include non-beneficiary households in their researches, thus relying on the beneficiary households to interpret their former situation, many times ending up with bias reporting, as the authors put it "respondents recall is often incorrect since it is hard to remember all past incidents correctly, resulting in over or under-reporting of past incidents". According to the submitted information, the authors have not published this study elsewhere. The structure of the paper is logical, starts with introducing the situation first and then presenting the methods and sources of data collection. In this case 188 households were included in the survey, cross-sectional survey data were gathered from these, further key informants interviews and focus group discussion was employed to triangulate results and a Chi-square test was employed to compare the households’ food security status. From the paper we can see that interviews and other analyses are performed to a high technical standard and are described in sufficient detail within the paper, the statistical analysis been performed appropriately and rigorously. The authors have made all data underlying the findings in their manuscript fully available within tables, explaining those tables as well. Also the conclusion is presented in the required way and data supports the findings, the policy implications are logical and supported by findings. The research meets all applicable standards for the ethics of experimentation and research integrity and the paper adheres to appropriate reporting guidelines and community standards for data availability. The only issue with the recent form of the paper is that it is not presented in an intelligible fashion and a language check is required as well. There are many typos within the text, missing spaces and the incorrect use of quotation marks (not where they are used but are cases where not the quotation mark is used for a citation or the citation is not closed correctly) make it harder to enjoy the paper. My recommendation is to publish the paper after the minor revisions regarding the correction of the language and style mistakes.

**Response:** The authors tried to improve the presentation styles and the language was also proofread by accredited editor.

Reviewer #3: The topic itself is really actual, even nowadays, despite the efforts of the country to deal with food secutity problems. The paper as a whole, the theme would be fine for publishing in PLOS ONE, however, in details, some modifications are needed.
- Introduction should be more focused and better structured (highlighting the research questions more clearly, context, objective)
- In fact, there is no literature review while it is a must. Authors should write a comprehensive, analytical and critical literature review chapter. In the paper the essential international literature dealing with this topic and/or focusing on Ethiopia should be reviewed. Other countres' cases could be interesting as well (e.g. <https://doi.org/10.21163%2FGT_2020.152.16> or the issue of trust is described well <https://doi.org/10.1080/08974438.2013.833567>)
- Metodology chapter should be better focused and more clear, now it seems like unstructured.
- English proofreading is needed to provide the expected scientific and right English.

**Response:** The authors tried to maintain the journal formalities and include the literature as suggested. The research questions and objectives are listed in a structured way.
Second time reviewers’ comments

Reviewer #1: The bibliographic extension was partially done, however it could be still further broadened, like <https://re.volsu.ru/eng/contacts/8_Nad_i_dr.pmd.pdf> , <https://ojs.lib.unideb.hu/apstract/article/view/6217/5834> .
The authors were also advised to specify and underline the applicability of their new results and findings in a structured list of recommendations, but it is still missing from the revised version. In my opinion, these improvements may merely increase the value of the paper. After the suggested recommendations were implemented the paper can be published.

**Response:** The authors appreciate the comments of the reviewer and tried to expand using recent published papers and reviewers suggested articles.

Reviewer #3: The authors did not made the recommended improvements so I can't accept this paper for publication in its present form. I advise to check my comments and recommendations of the first review round again.

**Response:** We revised based on the previous comments and suggestions. If we go beyond this, and add more literature, the page number will be increased and out of the PLOS ONE submission guideline.
