# Supplementary material for: Impact of productive safety net program on food security of beneficiary households in western Ethiopia: A matching estimator approach
Source: PLoS One. 2022 Jan 21;17(1):e0260817. doi: 10.1371/journal.pone.0260817 (PMC8782465; doi:10.1371/journal.pone.0260817)
Supplement: Attachment — Submitted filename: Response to reviewers November 2021.docx [file pone.0260817.s005.docx]

**Response to reviewer**

Reviewer #2: After reading the submission (the newly submitted version), though the authors claimed they have corrected everything, i still can see many mistakes which are, in my understanding, not acceptable for an academic journal. There are still many typos within the text, the ones which have been highlighted already (missing spaces and the incorrect use of quotation marks). These has to be corrected before acceptance.

**Response:** We tried to read seriously and address all the comments. See the track change for evidence
